# The Cholinergic Receptor Nicotinic α3 Was Reduced in the Hippocampus of Early Cognitively Impaired Adult Male Mice and Upregulated by Nicotine and Cytisine in HT22 Cells

**DOI:** 10.3390/cells14050340

**Published:** 2025-02-26

**Authors:** Hidetaka Ota, Takako Ohnuma, Ayuto Kodama, Tatsunori Shimizu, Kaoru Sugawara, Fumio Yamamoto

**Affiliations:** Advanced Research Center for Geriatric and Gerontology, Akita University, Akita 010-8543, Japan; ohnuma@med.akita-u.ac.jp (T.O.); ay-kodama@med.akita-u.ac.jp (A.K.); tatsunori@med.akita-u.ac.jp (T.S.); kaoruko02@jimu.akita-u.ac.jp (K.S.);

**Keywords:** early age-related cognitive decline, frailty, cholinergic receptor nicotinic α3 subunit, nicotine, cytisine

## Abstract

Ageing is a major risk factor for cognitive and physical decline, but its mechanisms remain poorly understood. This study aimed to detect early cognitive and physical changes, and to analyze the pathway involved by monitoring two groups of mice: a young and an adult group. The study has identified the types of molecules involved in the hippocampus. Adult mice (47 weeks) showed significantly reduced exploratory behavior compared to young mice (11 weeks), although spatial working memory showed no difference. In terms of physical function, grip strength was significantly reduced in adult mice. The Frailty Index (FI) further highlighted age-related changes in adult mice. To investigate the causes of cognitive decline, adult mice were categorized based on their declining cognitive function. Microarray analysis of their hippocampi revealed that the cholinergic receptor nicotinic α3 subunit (Chrna3) was significantly reduced in mice with cognitive decline compared to controls. Subsequent in vitro experiments showed that oxidative stress and cholinesterase inhibitors decreased Chrna3 expression, whereas nicotine and cytisine increased it. These results suggest that Chrna3 is a key factor in age-related cognitive decline. The development of therapeutic strategies targeting Chrna3 expression may offer promising avenues for preclinical and clinical research to mitigate cognitive ageing.

## 1. Introduction

Ageing has emerged as a significant societal concern globally, precipitating a myriad of health issues attributable to diverse factors [1]. It constitutes a major risk factor for diseases, including neurocognitive and physical impairments [2]. The global prevalence of dementia is estimated at 50 million, with projections suggesting an increase to 150 million by 2050 [3]. Preventing cognitive decline is deemed crucial, as much of the neuropathology associated with dementia accumulates progressively over 20–30 years prior to its clinical onset [4]. Physical frailty, characterized by increased vulnerability to a spectrum of age-related stressors in conjunction with cognitive decline, serves as an important indicator of health and risk variation among the elderly population [5]. Despite ongoing research endeavors aimed at elucidating the etiologies of these age-related conditions, their underlying mechanisms remain inadequately understood. Consequently, there is an urgent imperative to identify and enhance resilience in older adults, thereby facilitating the maintenance of independence and quality of life.

Recent research highlights the recognition of changes in motor performance in older adults as an early indicator of cognitive decline and dementia [6,7]. In our previous study, we demonstrated an association between slow walking speed and cognitive decline in older people [8]. This association may be due to common risk factors such as cardiovascular disease [9], diabetes [10], depression, and movement disorders [11], which are more likely to occur in older people. Alternatively, they could be the result of common neural pathways affected by cerebral small vessel disease [12,13] or Alzheimer’s disease (AD) pathology [14,15]. Current research focuses on the relationship between motor biomarkers and cognitive indicators. For example, slow walking and subjective cognitive complaints have been associated with the risk of developing dementia [16]. Thus, a number of neurophysiological markers have been proposed to detect early cognitive decline. However, for many specialists, there is no clear answer. These associated findings raise questions about what causes age-related cognitive and physical symptoms, and what mechanisms and molecules are involved.

In order to address these questions, we observed naturally ageing adult mice rather than models of AD, already senile dementia, and age-related frailty phenotypes. The aim of this study was to detect cognitive and physical changes early and to analyze the pathway involved by monitoring two groups of mice: a young group and an adult group. The cognitive and physical functions of ageing mice were monitored at different time intervals. Specifically, we aimed to observe the initial manifestation and subsequent changes in these functions as a result of ageing. It is plausible that some adult mice will exhibit cognitive decline at the same ages. By selecting adult mice with cognitive decline and comparing them without cognitive decline, we intended to investigate which molecular alterations occurred in the brain, with a particular focus on the hippocampus. Furthermore, this study explored several compounds that may modulate the expression of a molecule altered in vitro, thereby suggesting potential therapeutic implications.

## 2. Materials and Methods

### 2.1. Cells

HT-22 mouse hippocampal cells were purchased from Sigma-Aldrich Co. LLC (Darmstadt, Germany). HT-22 cells were cultured in DMEM high glucose (Sigma-Aldrich) containing 10% Fetal Bovine Serum.

### 2.2. Mouse Models

Animal experiments were carried out in accordance with the National Institute of Health Guide for the Care and Use of Laboratory Animals (8th edition, 2011), adopted by the research ethic committee of the faculty of medicine, Akita University, that approved the current protocol (a-1-0462, approval date: 16 February 2023). We used the C57BL/6NCrSlc strain, which is commonly employed in ageing research due to its well-documented genotypic and phenotypic characteristics [17]. Young and adult male mice were all housed and maintained in a room at 22 ± 2 °C with automatic light cycles (12 h light/dark) and a relative humidity of 40–60%. The mice were provided with a standard diet, 5L3R (PMI Nutrition International, Shoreview, MN, USA), and had access to fresh water at all times via an automated watering system. The cages were furnished with appropriate bedding materials to absorb moisture and provide comfort and were subjected to regular cleaning. These young mice of 4 weeks of age and adult mice of 40 weeks of age were purchased from Japan SLC, Inc. (Shizuoka, Japan). In the Y maze and open field test of this study, groups of male young (*n* = 20) of 4 weeks of age and adult (*n* = 20) of 40 weeks of age were first tested twice a month. A two-week interval was utilized to circumvent the carry-over effect that can ensue from the repetition of cognitive function tests. The point at which cognitive function began to decline in adult mice was at 47 weeks of age. Therefore, these mice were divided into the following three groups: A group of young mice with normal cognitive function (p1), a group of adult mice with normal cognitive function (p2), and a group of adult mice with cognitive function decline (p3). Mice were given T-scores and ranked based on their performance in cognitive function tests. The T-score was calculated using the standard formula, where each animal’s performance in the relevant tests was compared to the mean and standard deviation of the entire cohort. Specifically, the T-score for each animal was calculated using the following formula T = (X − μ)/σ, where X represents the individual animal’s score on a particular test, μ is the mean score of the entire group, and σ is the standard deviation. This standardized score allowed us to categorize animals based on their performance relative to the group as a whole. Based on G-power calculations, a minimum of 13 mice per group was required to achieve sufficient statistical power (80% or greater) to compare the three groups (p1, p2, p3). However, in this study we were limited to a total of 20 mice per group. In addition, the natural deaths of some mice during the study period made it impossible to achieve the sample size required for statistical evaluation. Therefore, we applied the principle of reduction to balance ethical considerations (principles of the 3Rs) since dissection was involved, and to maximize the data obtained from the available animals (*n* = 3). We continued to assess cognitive and physical function and presented data at 25 weeks of age for young mice and 61 weeks of age for adult mice.

### 2.3. Mouse Clinical FI Assessment

Mice were assessed for frailty using the 31-item mouse clinical FI, as described previously [18,19]. Briefly, young and adult mice were taken to a quiet room and allowed to acclimatize. The clinical assessment of deficits was then completed for each mouse; mice with no deficit received a score of 0, those with a mild deficit received a score of 0.5, and mice with a severe deficit received a score of 1. Values for each deficit were then summed and divided by the total number of deficits measured to yield an FI score theoretically between 0 and 1. Adult mice were assessed at 47 and 61 weeks of age compared with young mice at 11 and 25 weeks of age, and FI scores were balanced between young (p1) and adult groups (p2, p3) [20].

### 2.4. Physical Function Measurements

The forelimb grip strength of these mice was determined using a Grip Strength Meter (Columbus Instruments, Columbus, OH, USA). Grip strength was normalized by body weight. The locomotor functions (traveled distance, average walking speed) of these mice were assessed with open field tests.

### 2.5. Y-Maze Test

The Y-maze test (O’Hara Co., Ltd., Tokyo, Japan) was performed. This is a hippocampal-dependent short-term spatial working memory and reference memory test used to measure the willingness of rodents to explore new environments [21]. The behavior of young and adult mice, i.e., the entry times and positions of the arms, was observed for 5 min. These behavioral experiments were conducted during the dark phase, in accordance with the nocturnal nature of mice. When the mouse’s whole body was entered as an arm, the behavior was counted as an entry time. When the mouse entered three different arms successively, the alternation behavior was thought to reflect the capacity of working memory. The times of spontaneous alternation behavior was counted, and the ratio of alternation was calculated as ratio of alternation (times of spontaneous alternation/total times of arm entries-2) [20].

### 2.6. Open Field Test

The open field test fear response to novel stimuli was used to assess exploratory and anxiety-like behavior. Open field test protocols were modified [22]. The open field system was used (O’Hara Co., Ltd., Tokyo, Japan). A 10 cm area near the surrounding wall was delimited and considered the periphery. The rest of the open field was considered the central area [20]. The distance traveled, the ratio of the distance traveled in the central area/total distance traveled, and the time in the center of the open field were analyzed as measures of anxiety-like behavior. During the test, young and adult mice were allowed to move freely around the open field system and to explore the environment for 5 min.

### 2.7. Treatment with H_2_O_2_, Donepezil, Rivastigmine, Galantamine, Nicotine, Lobeline, Cytisine

HT-22 cells were grown in 100-mm collagen- or not-coated dishes to 80% confluence. They were treated for 0, 10, 20, and 30 min with 50 μmol/L and 100 μmol/L hydrogen peroxide (H_2_O_2_) diluted in a culture medium. HT-22 cells were treated with vehicle (0.1% ethanol), Donepezil (MedChemExpress Co., Monmouth Junction, NJ, USA), Rivastigmine (Combi-Blocks, Inc., San Diego, CA, USA), Galantamine (Cayman Chemical Co., An Arbor, MI, USA), Nicotine (MP Biomedicals, Inc., Irvine, CA, USA), Lobeline (MedChemExpress Co., Monmouth Junction, NJ, USA), Cytisine (ChemBrige Corporation, San Diego, CA, USA), at concentrations of 0, 5, 10, 20, and 25 μmol/L diluted in the medium for 72 h.

### 2.8. Microarray Analysis of Mice Brain Hippocampus

Mice were fully anesthetized and dissected after reflux with PBS to extract the brain. The hippocampus was extracted from the brain in a short time. RNA was extracted from hippocampus by RNeasy mini kits (QIAGEN Co., Hilden, Germany). The total RNA was isolated from each biopsy and individual samples were used for gene expression profiles. Target RNA was prepared by converting mRNA into double-stranded cDNA with a T7-(dT)_24_ primer incorporating a T7 RNA polymerase promoter. Double stranded cDNA synthesis and biotin-labeled cRNA was performed with a GeneChip^®^ WT Plus Kit (Affymetrix, Thermo Fisher Scientific, Santa Clara, CA, USA). After complementary RNA had been fragmented to sizes ranging from 35 to 200 bases by heating (35 min at 95 °C), 10 μg of RNA fragments were hybridized (16 h at 45 °C) to the Affymetrix Mouse Clariom S Array (Affymetrix, Thermo Fisher Scientific), which interrogates over 20,800 well-annotated genes. After hybridization, the gene chips were automatically washed and stained with streptavidin-phycoerythrin by the GeneCip Fluidis Station 450. The chips were scanned using the GeneChip Scanner 3000 7G (Affymetrix, Thermo Fisher Scientific, Inc.) [23]. Primary data analysis was performed with the Affymetrix Transcriptome Analysis Console (TAC) software ver 4.0.3 (Applied Biosystems) and was normalized and analyzed following the TAC user guide. Each analysis of variance was performed by one-way ANOVA. To determine the significance of differentially expressed genes, a cut-off for the fold change value ±1.5 and ** *p* < 0.05 was applied. Data were deposited in the National Center for Biotechnology Information Gene Expression Omnibus (accession number: GSE272324).

### 2.9. Histological Analysis of Mice Hippocampus

Mice were fully anesthetized and dissected after perfusion with PBS to extract brain. They were then embedded in tissue-tek O.C.T compound (Sakura Finetek Japan, Ltd., Tokyo, Japan) to prepare frozen sections. DAPI (4′,6-Diamidino-2-phenylindole Dihydrochloride Solution) staining (Dojindo Molecular Technologies, Inc., Kumamoto, Japan) of the hippocampus was performed to detect the cell nucleus. The secondary antibodies (Alexa Fluor 488 donkey) and antifade reagent were from Molecular Probes (Invitrogen). Fluorescent images were analyzed using a fluorescence microscope (BZ-X810, BZ-X800 analyzer, KEYENCE, Osaka, Japan).

### 2.10. Antibodies and Immunoblotting

HT-22 cells were lysed on ice for 1 h in buffer (50 mM Tris–HCl, pH 7.6, 150 mM NaCl, 1% NP-40, 0.1% SDS, 1 mM dithiothreitol, 1 mM sodiumvanadate, 1 mM phenylmethylsulfonylfluoride, 10 μg/mL aprotinin,10 μg/mL leupeptin and 10 mM sodiumfluoride). After blocking, the filters were incubated with the following antibodies: anti-Chrna3 (PA5-116458, Thermo Fisher Scientific) and anti-ß-actin (Cell Signaling Technology, Inc., Danvers, MA, USA). After washing and incubation with horseradish peroxidase-conjugated anti-rabbit IgG (Cell Signaling Technology, Inc.) for 1 h, the antigen-antibody complexes were visualized using an enhanced chemiluminescence system (Bio Rad Laboratories, Inc., Hercules, CA, USA).

### 2.11. Viability Analysis of HT-22 Cells

The viability (%) of control vehicle (0.1% ethanol), H_2_O_2_ 50 μM, 100 μM treated HT-22 cells were analyzed after 10–30 min treatment. 10 μL of these cell suspensions were added to 10 μL of 0.4% trypan blue solution and mixed gently by pipetting. Then, 10 μL of the mixture was pipetted and loaded into the counting chamber. Each cell count and viability were assessed using a TC20 automated cell counter (Biorad Laboratories, Inc., Boulder, CO, USA) [20].

### 2.12. Statistics

The results of in vivo and vitro studies were expressed as mean ± standard deviations (SD). Student’s *t*-test, unpaired *t*-test, and one-way ANOVA were used to perform the comparison of statistics among a set of samples with SPSS (Version 26.0, SPSS, Inc., Chicago, IL, USA). * *p* < 0.01 and ** *p* < 0.05 were considered a significant difference.

## 3. Results

### 3.1. Comparison of Cognitive and Physical Function of Young and Adult Mice

**Figure 1:**
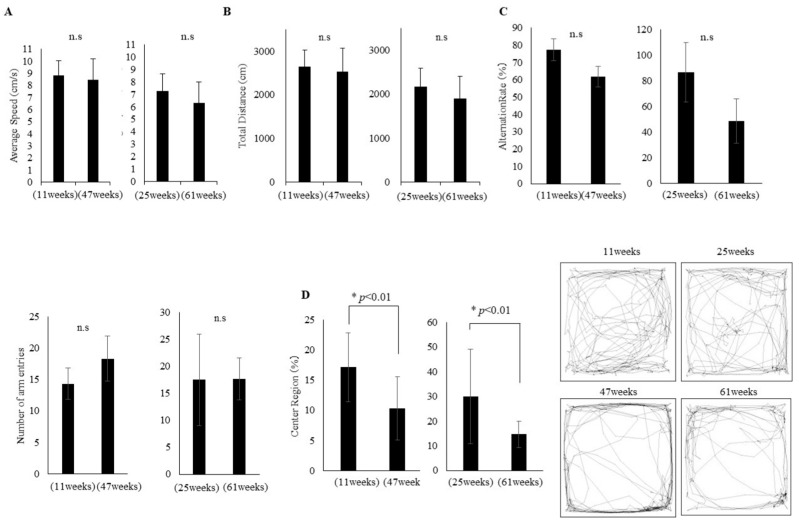
Comparison of cognitive and physical function of young and adult mice. (**A**) Comparison of average speed (cm/s) in young (11, 25 weeks, *n* = 20, 17) and adult (47, 61 weeks, *n* = 20, 14) mice. (**B**) Comparison of total traveled distance (cm) in (11, 25 weeks) and adult (47, 61 weeks) mice. (**C**) Percentage (%) of spontaneous alternations and total number of arm entries in Y maze test, and (**D**) Central region/total distance (%) in open field test in young (11, 25 weeks) and adult mice (47, 61 weeks). The traveled paths in the open field test in representative young (**top**) and representative old mouse (**bottom**) shown at the right panel. The number of mice utilized in these experiments was reduced (young group: 3 mice; adult group: 6 mice). All cases of dropout were due to death without prodromal symptoms. Unpaired *t*-test, * *p* < 0.01, Mean ± SD, n.s: not significant.

In this study, mice (C57BL/6NCrSlc) were assessed every two weeks for cognitive and physical functioning, starting at 4 and 40 weeks of age, for 21 weeks. A longitudinal analysis revealed significant differences between them at 11 and 47 weeks of age. No differences were observed between the young (11 weeks) and adult (47 weeks) groups with regard to physical function tests, including average usual walking speed (Figure 1A) and total distance traveled (Figure 1B). Furthermore, no differences were observed between the two groups in the Y-maze test, which is a test of spatial working memory function (Figure 1C). In the open field test, the percentage of time spent in the center of the open field area is used as an indicator of anxiety-like behavior. Lower percentages indicate higher levels of anxiety, as animals tend to avoid the center due to their natural tendency to stay close to the walls (thigmotaxis). Conversely, it was observed that anxiety-like behavior was significantly higher in adult mice than in young mice (Figure 1D). Further observation continued over time until the young mice were 25 weeks of age and the adult mice were 61 weeks of age. Consequently, no significant difference in physical functions was observed between the two groups (Figure 1A,B). However, in terms of cognitive function tests, although no significant difference was found in spatial working memory and spontaneous locomotor activity (Figure 1C), there was a decline in exploratory behavior in adult mice (61 weeks) compared to young mice (25 weeks) (Figure 1D).

**Figure 2:**
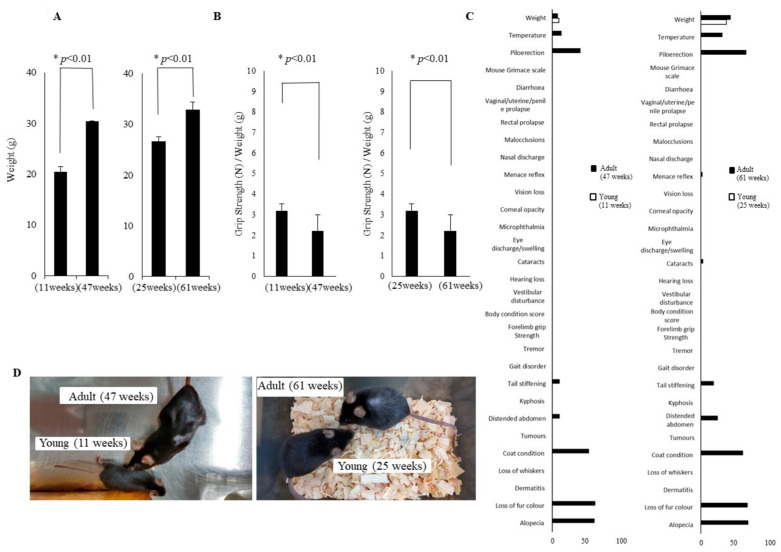
Weight, grip strength, and FI measurements in young and adult mice. (**A**) Differences in body weight (g), (**B**) normalized grip strength (N) by weight in young (11 weeks, *n* = 20), (25 weeks, *n* = 17) and adult (47 weeks, *n* = 20), (61 weeks, *n* = 14) mice. (**C**) A range proportion (%) of individual FI deficits of young (11, 25 weeks, white) and adult (47, 61 weeks, black) mice are shown. (**D**) Appearance of 11 weeks and 47 weeks of age mice (right photo), 25 weeks and 61 weeks of age mice (left photo) are shown. The number of mice utilized in these experiments was reduced (young group: 3 mice; adult group: 6 mice). All cases of dropout were due to death without prodromal symptoms. Unpaired *t*-test, * *p* < 0.01, Mean ± SD.

In addition to assessing cognitive and physical function, we also evaluated the frailty phenotype. In terms of body weight, young mice at 11 weeks of age exhibited a significantly lower weight than adult mice at 47 weeks of age. However, the difference in body weight between young mice at 25 weeks of age and adult mice at 61 weeks of age was significantly lower, and the weight gain rate of the young mice increased (Figure 2A). Grip strength was also evaluated. A comparison of young mice at 11 weeks of age with adult mice at 47 weeks of age revealed a significant reduction in grip strength in the latter (Figure 2B). This trend remained consistent as the mice aged further to 25 and 61 weeks of age (Figure 2B). Subsequently, in order to assess the frailty phenotype in a comprehensive manner, we employed the FI, which is a widely utilized instrument in animal experimentation. A comparison of young mice at 11 weeks of age and adult mice at 47 weeks of age revealed that several physiological indicators of ageing were evident, including body temperature, piloerection, tail stiffening, coat condition, loss of coat color and alopecia (Figure 2C,D). Similarly, further indications of frailty were observed in young mice at 25 weeks of age and adult mice at 61 weeks of age, and phenomena such as cataracts and threat reflex were also observed (Figure 2C,D).

### 3.2. Comparison of Young Mice (p1), Adult Mice with Normal (p2), and with Declining Cognition (p3)

**Figure 3:**
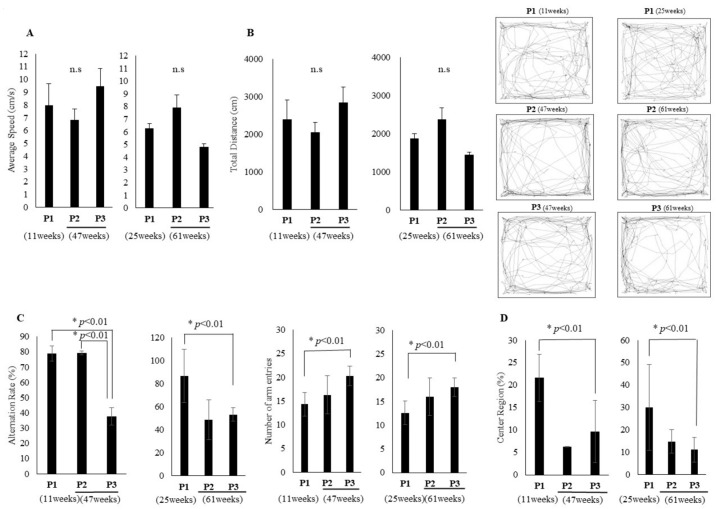
Comparison of young mice (p1), adult mice with normal (p2), and with declining cognition (p3). The mice were divided into three groups: young mice with normal cognitive function (p1, 11, 25 weeks of age, *n* = 3), adult mice with normal cognitive function (p2, 47, 61 weeks of age, *n* = 3), and adult mice with cognitive function declined (p3, 47, 61 weeks of age, *n* = 3). (**A**) Comparison of average speed (cm/s), (**B**) Total traveled distance (cm) in p1, p2, and p3 groups. (**C**) Percentage (%) of spontaneous alternations and total number of arm entries in Y maze test. (**D**) Central region/total distance in open field test in p1, p2, and p3 groups. The traveled paths in the open field test in representative p1, p2, and p3 (left: 11 vs. 47 weeks, right: 25 vs. 61 weeks) representatives are shown at the right panel. Unpaired *t*-test, * *p* < 0.01, Mean ± SD, n.s: not significant.

**Figure 4:**
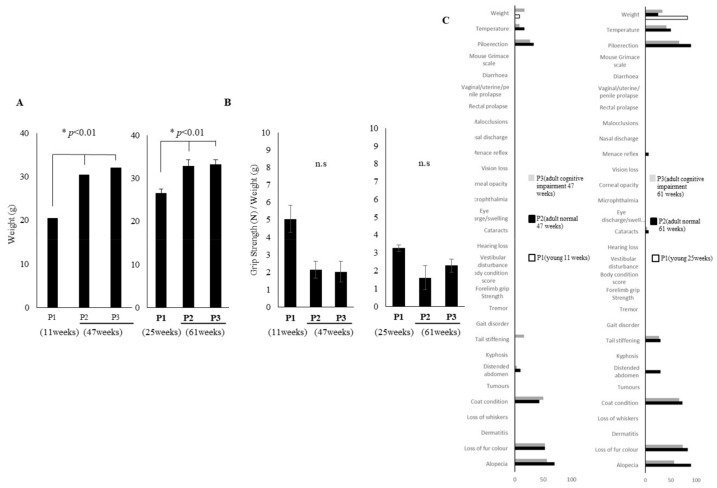
Weight, grip strength, and FI measurements in young mice (p1), adult mice with cognitive normal (p2) and decline (p3). (**A**) Differences in body weight (g), (**B**) Normalized grip strength (N) by weight in p1, p2, and p3 groups (*n* = 3). (**C**) A range proportion (%) of individual FI deficits of p1 (white), p2 (grey), and p3 (black) groups are shown. Unpaired *t*-test, * *p* < 0.01, Mean ± SD, n.s: not significant.

In order to ascertain the cause of early age-related cognitive decline, we selected adult mice with declining cognitive function. The selection method involved comprehensively ranking the T-scores of adult mice based on the results of two cognitive function tests (Y-maze test, open field test). The lowest three mice were then selected in accordance with the principles of the 3R (*n* = 3). The mice were then divided into three groups: young mice with normal cognitive function (p1), adult mice with normal cognitive function (p2), and adult mice with cognitive function decline (p3). The aim of reassessing subgroup performance was to gain a clearer understanding of the molecular mechanisms underlying different cognitive functions and to ensure that molecular differences consistently correlated with subgroup performance differences and were not simply artefacts of the initial test results. No significant difference was observed between the three groups (p1–p3) of young mice (11 weeks) and adult mice (47 weeks) with respect to average walking speed. Similar results were obtained for young mice (25 weeks) and adult mice (61 weeks) (Figure 3A). With regard to the total walking distance, no significant difference was observed between the three groups (p1–p3): young mice (11 weeks) and old mice (47 weeks), and young mice (25 weeks) and adult mice (61 weeks) (Figure 3B). In the Y-maze test, when comparing three groups (p1–p3) of young mice at 11 weeks of age and adult mice at 47 weeks of age, the p3 group demonstrated a significant difference compared to the p1 and p2 groups. It was confirmed that spatial working memory was significantly reduced in p3 (Figure 3C). A comparison of three groups of young (25 weeks) and adult (61 weeks) mice revealed a significant decrease in the p3 group compared to the p1. A similar difference was observed when the p3 was compared with the p2 (Figure 3C). No significant difference was found in spontaneous locomotor activity (Figure 3C). Furthermore, when comparing three groups of young (11 weeks) and adult (47 weeks) mice in an open field test, the p3 group demonstrated reduced exploratory behavior compared to the p1 group. However, no significant difference was observed between the p3 and p2 groups (Figure 3D). When comparing three groups of young (25 weeks) and adult (61 weeks) mice, there was a tendency for the p3 group to exhibit lower exploratory behavior than the p1 and p2 groups (Figure 3D). The mice were then assessed for frailty. Body weights were compared for three groups of young mice (11, 25 weeks) and adult mice (47, 61 weeks). The results demonstrated that p2 and p3 were significantly heavier than p1 (Figure 4A). No significant difference in grip strength was observed between the three groups (Figure 4B). Finally, the ageing phenotype was assessed using the FI. Consequently, both the p2 and p3 exhibited significantly accelerated ageing symptoms in comparison to the p1 (Figure 4C).

### 3.3. Chrna3 Expression Was Reduced in the Hippocampus of Adult Mice with Cognitive Decline Compared to Adult Mice Without Cognitive Decline

**Figure 5:**
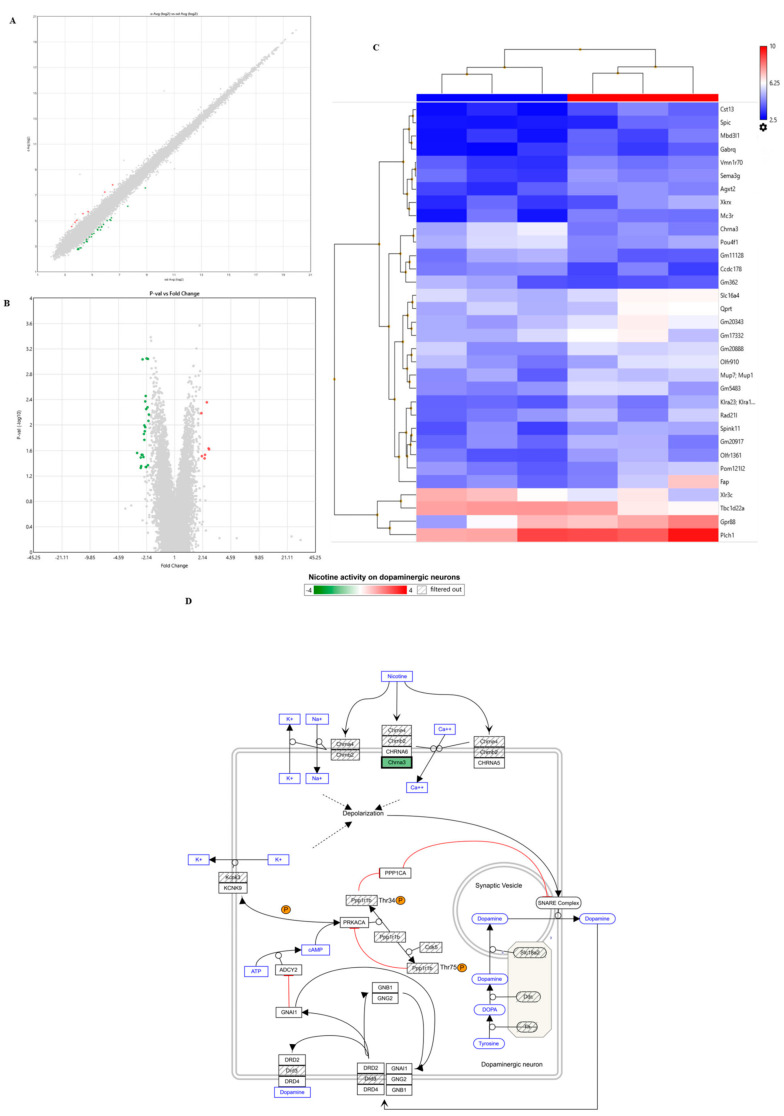
DNA microanalysis of adult mice with cognitive normal (p2) and decline (p3) in hippocampus. (**A**) Scatter plots, (**B**) volcano plots, and (**C**) hierarchical clustering compared with p2 and p3 groups. Red plots showed upregulation and green plots showed downregulation. (**D**) Signaling analysis pathway of Chrna3. Primary data analysis was performed with TAC software and was normalized and analyzed following the TAC user guide. Each analysis of variance was performed by one-way ANOVA. To determine the significance of differentially expressed genes, a cut-off for the fold change value ±1.5. Data were deposited in the National Center for Biotechnology Information Gene Expression Omnibus (accession number: GSE272324).

In this study, we focused our analysis on the hippocampus, which plays an important role in maintaining memory function. We studied adult mice at 47 weeks of age, when signs of changes in cognitive function were first observed, and young mice at 11 weeks of age. From these three groups (p1–p3) of mice, the hippocampus was removed from the whole brain and RNA was extracted. The RNA was converted to cDNA and DNA microarray analysis was performed to compare differences in gene expression. When comparing a group of adult mice with normal cognition (p2) and a group of adult mice with cognitive decline (p3), changes in the expression of approximately 170–230 genes were observed (Figure 5A,B). Among the various genes whose expressions changed, significant changes were observed in genes related to cognitive functions, with the expression of Chrna3 being detected as the most significant change (Figure 5C). We then compared a group of young mice with normal cognitive function (p1) and a group of adult mice with normal cognitive function (p2). Changes were observed in approximately 150–190 gene clusters. The gene cluster mainly contained genes related to inflammatory pathways such as cytokines and complement, and metabolic pathways related to insulin, cytoskeleton, and hormones. Among these, various factors such as insulin signaling, which is involved in metabolic pathways, have been reported to play multifaceted roles in cognitive ability [24], but genes directly related to neural signals were not significantly detected. Finally, changes in Chrna3 expression were confirmed by immunohistochemistry. We found that Chrna3 expression was significantly reduced in adult mice with cognitive decline compared to adult mice without cognitive decline (Supplementary Figure S1).

### 3.4. Chrna3 Expression Was Downregulated by Oxidative Stress and Cholinesterase Inhibitors, and Upregulated by Nicotine and Cytisine in HT-22 Cells

**Figure 6:**
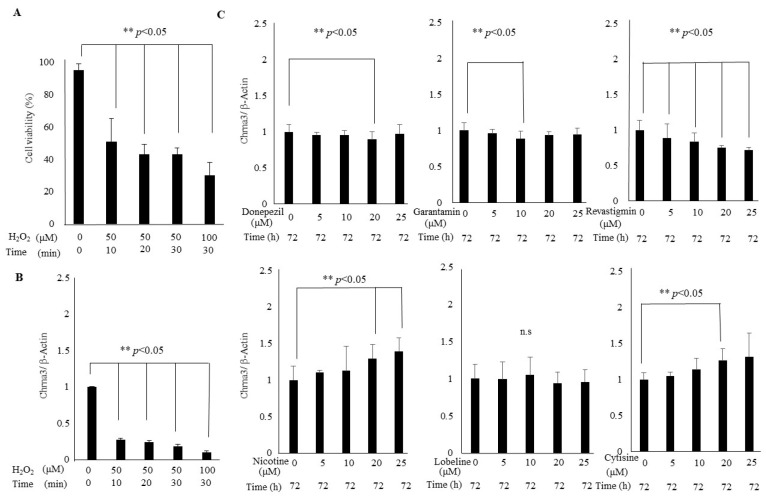
Chrna3 expression treated HT-22 cells with hydrogen peroxide, donepezil, rivastigmine, galantamine, nicotine, lobeline, and cytisine. (**A**) Viability (%) HT-22 cells of H_2_O_2_ control vehicle (0.1% ethanol), H_2_O_2_ treatment (50, 100 μM) for 0, 10, 20, and 30 min. (**B**) Immunoblotting intensity of control vehicle, H_2_O_2_ (50, 100 μM) treated HT-22 cells using Chrna3 and β-actin antibody. (**C**) Immunoblotting intensity of control vehicle, donepezil, rivastigmine, galantamine, nicotine, lobeline, and cytisine (0, 5, 10, 20, and 25 μmol/L) for 72 h treated HT-22 cells using Chrna3 and β-actin antibody. Repeated experiments were performed (*n* = 3) and representative data shown. Student’s *t*-test, ** *p* < 0.05, n.s: not significant.

Based on the results of these animal experiments, we then investigated how Chrna3 expression changes in response to various exogenous stimuli in vitro. We carried out in vitro experiments using HT-22 cells, which are derived from mouse hippocampal cells. Since oxidative stress is one of the major stress factors promoting the ageing process, we first decided to treat the HT-22 cells with H_2_O_2_. After H_2_O_2_ treatment for 0, 10, 20, and 30 min, the cell viability (%) of HT-22 cells was found to decrease in a time- and concentration-dependent manner compared to untreated controls (Figure 6A). Furthermore, Chrna3 expression was found to be similarly decreased by oxidative stress compared to the control (Figure 6B).

Furthermore, we observed chemical compounds that alter Chrna3 expression. First, we considered as candidate compounds those which are used clinically to treat AD, particularly to improve cognitive function. HT-22 cells were treated with donepezil, rivastigmine, and galantamine for 72 h, which are cholinesterase inhibitors that increase acetylcholine [25]. It was observed that treatment with donepezil and galantamine decreased Chrna3 expression at the concentration of 20 and 10 μM respectively, and rivastigmine showed a dose-dependent decrease (Figure 6C). We have also had access to the database for compounds that change Chrna3 expression. The database used in this study was the KEGG (Kyoto Encyclopedia of Genes and Genomes) [26], a database that integrates information on intermolecular networks of genes, proteins, metabolism, and signaling. According to this database, 18 compounds targeting Chrna3 were identified [27]. Most of these compounds were related to the musculoskeletal system, but these were eliminated and those acting directly on the central nervous system were selected, with the exception of acetylcholine. As a result, nicotine, cytisine, and lobeline were selected as candidates. Cytisine and lobeline are alkaloids that occur naturally in several plants and act as nicotinic agonists [28]. When these compounds were treated in HT-22 cells, lobeline did not alter Chrna3 expression. On the other hand, nicotine and cytisine were observed to significantly increase Chrna3 expression in a concentration-dependent manner (Figure 6C).

## 4. Discussion

Given that ageing is the major risk factor for dementia [29] and frailty [30], we conducted longitudinal observations of naturally ageing mice to determine the sequence and extent of early onset of cognitive and physical functional decline and to identify the related molecular changes. Mice aged 4 and 40 weeks were monitored over time, and it was at 47 weeks of age that the first signs of cognitive decline were observed. The initial decline was observed in exploratory behavior (Figure 1D). In terms of physical function, no changes in usual walking speed or total distance traveled, which reflect lower limb muscle strength, were observed during the observation period (Figure 1A,B). On the other hand, changes in body weight and grip strength, which reflect upper limb muscle strength, were observed (Figure 2B). Numerous neurophysiological markers have been proposed to detect early cognitive impairment, yet diagnosis remains challenging for specialists [31]. Our findings indicate that cognitive decline initially manifests in motivation-related functions such as exploratory and anxiety-like behavior, followed by a decline in cognitive functions such as spatial working memory. Additionally, in terms of physical function, our results showed that weight increases with age and muscle strength decreases faster in the upper limb than in the lower limbs. Although recent studies have highlighted usual walking speed as a biomarker for early detection of cognitive decline [32], our results suggest that upper limb muscle strength is equally important. Indeed, previous work has reported associations between upper limb muscle weakness and cognitive function [33,34]. Moreover, when assessing the ageing phenotype using FI, middle-aged mice exhibited higher body temperature, piloerection, hair loss, loss of coat color, abdominal distension, and cataracts compared to younger mice (Figure 2C,D).

Similar to humans, some ageing mice maintain cognitive function while others do not. To understand why cognitive function varies among mice of the same age, we hypothesized that comparing these two groups of adult mice (p2, p3), while using young mice as controls (p1), would reveal the underlying causes of early cognitive decline. The selection method involved ranking the T-scores of adult mice based on two cognitive function tests (Y-maze test, open field test) and selecting the three lowest (p3) and three highest (p2) scoring mice, in accordance with the principles of the 3R (*n* = 3). Interestingly, when these selected mice were compared, the physical functions, i.e., usual walking speed, total traveled distance, grip strength, body weight, and FI tended to be similar to the results of all comparisons between young and adult mice (Figure 3A–C and Figure 4A–C), whereas the cognitive functions of adult mice (p2 vs. p3) showed a clear trend towards a reduction in spatial working memory rather than in exploratory behavior (Figure 3C,D).

Since the hippocampus is crucial for memory function, we extracted the hippocampi from these mice and used DNA microarrays to compare gene expression between cognitively normal and impaired adult mice (p2 vs. p3), and between young and adult mice (p1 vs. p2). The results showed a significant reduction in Chrna3 expression in cognitively impaired adult mice compared to those without decline (Figure 5C). This change in Chrna3 expression was not observed when comparing young and adult mice, indicating that it is not simply an effect of age differences. In the brain, nicotinic acetylcholine receptors (nAChRs) are widely expressed [35] and involved in regulating several functions, including cognitive and physical abilities. The nAChRs are Ach-activated cationic channels composed of nine α (α2–α10) and three β subunits (β2–β4) [36,37,38]. The homomeric (α7 or α9) or heterometric (α2–α6 with β2–β4) assembly of five subunits generates multiple subtypes with distinct pharmacological and functional properties [39]. The most widely expressed neuronal subtypes in the brain are heteromeric α4β2 and homomeric α7 receptors, whereas α3β4 is the most widely expressed subtype in the peripheral nervous system [40]. Although Chrna3 is not the most abundantly expressed nicotinic acetylcholine receptor (nAChR) subunit in the central nervous system, our results suggest that its expression was reduced in the hippocampus with cognitive decline. To investigate this further, we now examined the expression of other nAChR subunits in our data set. Our analysis showed that other specific subunits (e.g., Chrna4, Chrna7) did not change with age in the hippocampus, suggesting that age-related changes in the composition of other nAChRs may not influence cognitive function during ageing. In addition, different nAChR subtypes involved in α3β4 subunit have been reported in the hippocampus [41]. Despite unique functional properties, the subtypes overlap sufficiently, complicating their distinction using pharmacological agents [42]. In particular, the activation of Chrna3 suppresses anxiety behavior [43]. It is also known that the activation of Chrna3 improves cognitive functions such as memory and attention, while a reduction in Chrna3 leads to a decline in cognitive function [44]. Thus, Chrna3 is deeply involved in the regulation of important neural functions such as anxiety, cognition, and reward, and it is becoming clear that changes in its expression level and activity are involved in the pathology of various psychiatric and neurological disorders.

We then investigated how Chrna3 expression responds to exogenous stimuli using HT22 cells, derived from the mouse hippocampus. First, we examined whether age-related stress affects its expression. Oxidative stress, used as an age-related stress model [45], reduced Chrna3 expression in a time- and concentration-dependent manner (Figure 6B). These results suggest that increased oxidative stress may decrease Chrna3 expression, contributing to cognitive decline. It is well known that nAChRs are targets of the neurotransmitter acetylcholine, nicotine, and many high-affinity nicotinic agonists [46]. It has been reported that hippocampal changes induced by overexpression of the human Chrna3 gene cluster may underlie cognitive deficits rescued by nicotine in transgenic mice [47]. Previous reports have demonstrated that treatment with cholinesterase inhibitors increase the expression of the α4 subunit at the cellular level [48]. Therefore, we treated with donepezil, rivastigmine, and galantamine. However, contrary to our expectations, treatment with them did not change Chrna3 expression and, on the contrary, decreased it (Figure 6C). Finally, using the KEGG database, nicotine, cytisine and lobeline were identified as potential candidates [27]. Treatment with nicotine and cytisine increased Chrna3 expression in a dose-dependent manner, but lobeline had no effect (Figure 6C). Nicotine, acting via nAChRs, has shown short-term cognitive improvements, particularly in attention, working memory, and executive function [49,50], and has been found to ameliorate cognitive impairment in disease-related cognitive disorders such as age-related memory impairment, AD, Parkinson’s disease, schizophrenia, stroke, and attention deficit hyperactivity disorder [51]. Cytisine, a natural plant alkaloid used in smoking cessation, binds to nAchR and has been shown to reduce cognitive decline in the early stage of AD model mice [52]. The mechanisms by which nicotine and cytisine improve cognitive function are not fully understood, and there are no reports demonstrating that they upregulate Chrna3 expression specifically, as shown in this study. Recent studies indicate that α7 and Chrna3 expression are regulated by different mechanisms, with Chrna3 expression increasing upon Tyr kinase inhibition or Ser/Thr phosphatase 2A activation [53]. Our results showed that cholinesterase inhibitors decreased Chrna3 expression, whereas the direct agonists nicotine and cytisine increased it. There is currently no literature that clearly identifies these opposing mechanisms of Chrna3, and most studies have generally examined their effects on nAChRs. We speculate that cholinesterase inhibitors may suppress receptor gene transcription through sustained stimulation by elevated acetylcholine levels in the synaptic cleft. Conversely, nicotine and cytisine have been shown to increase receptor expression in animal models and smokers, and the mechanisms behind this have been speculated to be receptor aggregation, proteosomal degradation, trafficking, and cell surface expression [54,55]. Further experimental investigation is needed to better understand these different effects. Despite cardiotoxic side effects preventing the approval of several α7nAChR agonists, nicotine-derived agonistic compounds like cytisine, which increase Chrna3 expression, appear promising for cognitive function and neurological disease-related cognitive impairment.

A limitation of this study is the reliance on the Y-maze [56,57,58] and open field tests to assess cognitive and motor function, which require the inclusion of additional tests of hippocampal function (such as the Barnes maze, Morris water maze, etc.), activity measurements, or motor function (such as the rotarod, beam walking, etc.) and rigorous mouse screening. In addition, because our primary focus is on the behavioral measures, specifically the time spent in the center and the total distance traveled, we chose not to include fecal boli counts in the open field test. Moreover, the decision to use only male animals was primarily based on several factors. Firstly, male animals are often chosen to minimize variability, particularly in studies where hormonal fluctuations in females could introduce additional complexity and potentially confound the results. This is particularly important in longitudinal studies where consistency across time points is essential. In this study, we demonstrated that Chrna3 expression declines with age; however, we have not yet established Chrna3 as a key regulator of cognitive function in mice. We recognize that this approach has limitations and may not fully capture the broader applicability of our findings. Going forward, we plan to include female subjects in future studies to ensure a more comprehensive understanding of the phenomena we are investigating. Furthermore, the potential involvement of the cholinergic system in linking cognitive and physical functions suggests the need for muscle analysis. Although other studies have demonstrated the effects of these compounds on cognitive function, in the near future the effects of treating these compounds with cognitively impaired mice should be examined. Additionally, the potential toxic effects and side effects of nicotine and cytisine need to be thoroughly investigated.

## 5. Conclusions

In conclusion, this study, by longitudinally observing young and adult mice, found an initial decline in exploratory behavior, followed by a decrease in upper limb grip strength and physical frailty. It was also demonstrated that reduced Chrna3 expression may be involved in cognitive decline among adult mice, and that nicotine and cytisine could inhibit this decline. The findings suggest that future development strategies targeting Chrna3 expression are critical, particularly considering cytisine’s high affinity for nAChRs, lower addiction potential compared to nicotine, and safer use profile in preclinical and clinical trials.

## Data Availability

Microarray data were deposited in the National Center for Biotechnology Information Gene Expression Omnibus (https://www.ncbi.nlm.nih.gov/geo/ (accessed on 21 January 2025)). accession number: GSE272324.

## Supplementary Materials

The following supporting information can be downloaded at: https://www.mdpi.com/article/10.3390/cells14050340/s1, Figure S1: Chrna3 expression of young (p1), adult mice with cognitive normal (p2), and decline (p3) in hippocampus.

## Abbreviations

The following abbreviations are used in this manuscript:

AD	Alzheimer Disease
ANOVA	Analysis Of Variance
Chrna3	Cholinergic receptor nicotinic α3 subunit
DAPI	4′,6-Diamidino-2-phenylindole Dihydrochloride Solution
DNA	Deoxyribonucleotidic Acid
FI	Frailty Index
H_2_O_2_	Hydrogen Peroxide
KEGG	Kyoto Encyclopedia of Genes and Genomes
nAChR	nicotinic Acetylcholine Receptor
RNA	Ribonucleic Acid
SD	Standard Deviation
TAC	Transcriptome Analysis Console
