# Supplementary figures and images for: The Cholinergic Receptor Nicotinic α3 Was Reduced in the Hippocampus of Early Cognitively Impaired Adult Male Mice and Upregulated by Nicotine and Cytisine in HT22 Cells
Source: Cells. 2025 Feb 26;14(5):340. doi: 10.3390/cells14050340 (PMC11898884; doi:10.3390/cells14050340)

Supplementary Figure S1

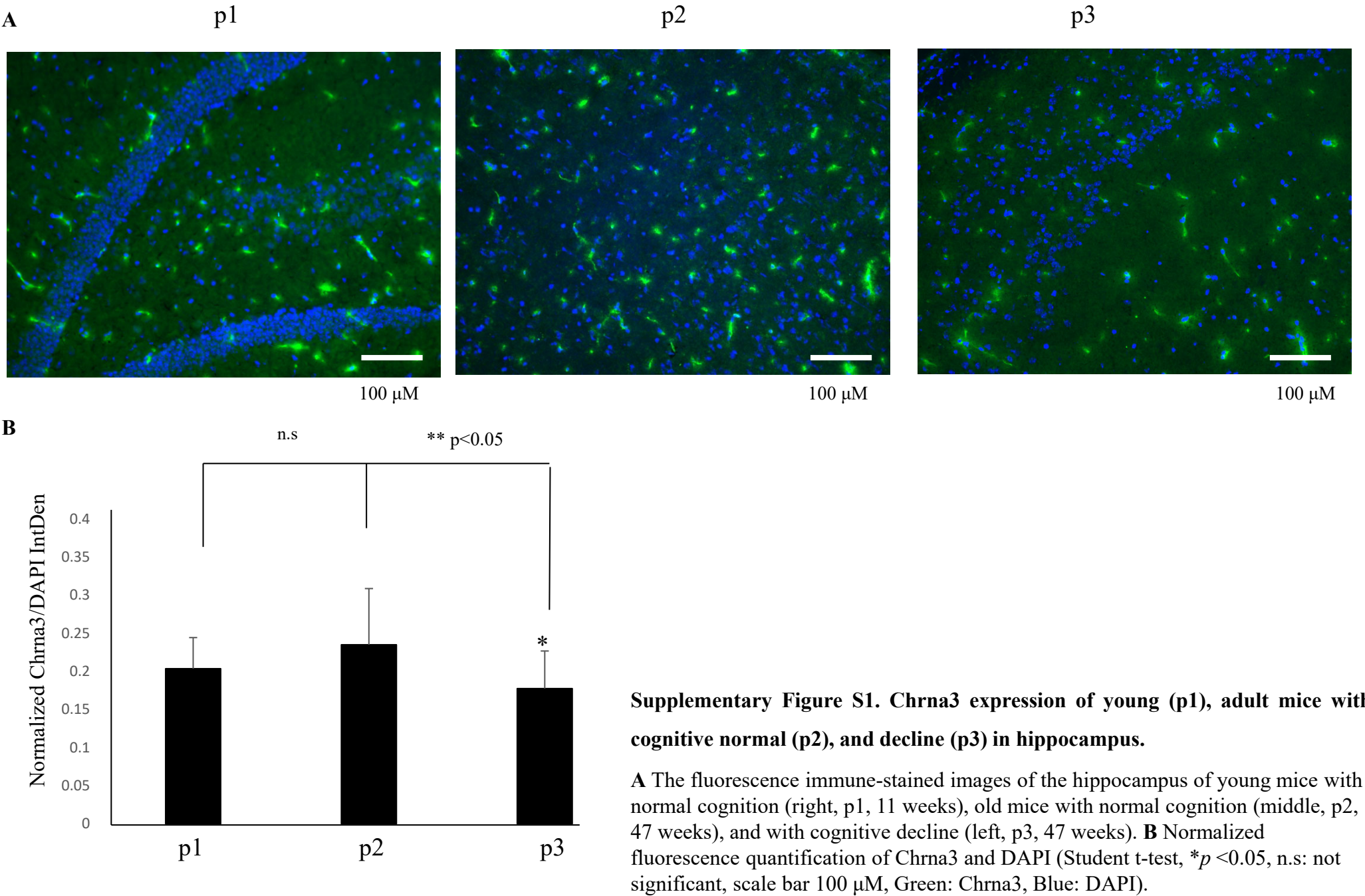

Supplement: Supplementary file 1 [file cells-14-00340-s001.zip › cells-3467545-supplementary.pdf]
